# Evaluating Open Dialogue in Italian mental health services: evidence from a multisite prospective cohort study

**DOI:** 10.3389/fpsyg.2024.1428689

**Published:** 2024-09-06

**Authors:** Raffaella Pocobello, Francesca Camilli, Pina Ridente, Giuseppa Caloro, Maria Giuseppe Balice, Giuseppe Tibaldi, Marcello Macario, Marco d’Alema, Elisa Gulino, Tarek el Sehity

**Affiliations:** ^1^Institute of Cognitive Sciences and Technologies, CNR, Rome, Italy; ^2^Azienda Sanitaria Universitaria Giuliano Isontina (ASU GI), Trieste, Italy; ^3^Dipartimento di Salute Mentale e Dipendenze Patologiche - AUSL Modena, Modena, Italy; ^4^DSM - ASL Città di Torino, Torino, Italy; ^5^Dipartimento Salute Mentale e Dipendenze, ASL 2, Savona, Italy; ^6^Dipartimento Salute Mentale e Dipendenze patologiche, Asl Roma 6, Rome, Italy; ^7^Modulo Dipartimentale Salute Mentale 1 Calatino, Azienda Sanitaria Provinciale di Catania, Catania, Italy; ^8^Faculty of Psychology, Sigmund Freud Private University, Vienna, Austria

**Keywords:** Open Dialogue, mental health, treatment outcomes, clinical outcomes, social networks, patient satisfaction

## Abstract

**Objective:**

This longitudinal study aimed to quantitatively document and evaluate the implementation and outcomes of the Open Dialogue (OD) approach within Italian Mental Health Departments (MHDs), focusing on the ratings of OD-network meetings by patients and their families and assessing the clinical outcomes over a span of 12 months.

**Results:**

Over the course of the study, 58 patients participated in 517 OD-network meetings, demonstrating a high level of satisfaction with the care received, as evidenced by the Session Rating Scale (SRS) and Outcome Rating Scale (ORS). Clinically, significant improvements were observed in the Global Assessment of Functioning (GAF), Clinical Outcomes in Routine Evaluation (CORE-OM), and the Lubben Social Network Scale (LSNS), indicating enhanced psychological and social functioning. The SRS scores showed that satisfaction with the meetings increased over time, while the ORS indicated that both patients and their social networks perceived gradual improvements throughout the therapy.

**Conclusion:**

The OD approach within Italian MHDs was successfully implemented and well-received by patients and their social networks, yielding significant clinical improvements. These findings suggest the feasibility and effectiveness of integrating the OD model into the Italian public mental health system, supporting its potential for broader application in diverse healthcare settings. The study highlights the importance of continuous engagement and evaluation to maintain high standards of practice and suggests that OD can be a valuable addition to existing mental health care practices, promoting recovery through inclusive, dialogue-based interventions.

## 1 Introduction

### 1.1 Background and principles of Open Dialogue

The public mental health service in the Finnish province of Western Lapland currently operates according to the principles of Open Dialogue (OD), an approach to mental health that emerged in the same area in the 1980s. Two main ideas are based on OD: one refers to the therapeutic approach that is adopted during meetings with patients, and the other applies to how the mental health service is organized (Seikkula et al., 2001).

According to the OD approach, treatment is provided in the form of “network meetings,” which include the participation of the patients, their family, their social network, and the crisis intervention team. Over the years, seven principles have been formalized to describe the characteristics of OD, with “tolerance of uncertainty” and “dialogism” being the two main guidelines at the base of the conversations that take place during the meetings (Seikkula et al., 2001). The first organizational principle refers to arranging the initial meeting 24 h after the first contact. The second organizational principle explains that the client’s social network, including family members and other key persons, must be invited to the first meeting with the client. “Flexibility and mobility,” the third organizational principle, deals with the idea that treatment should adapt to the client’s needs, and the meetings should be arranged as much as possible at their home. According to the fourth organizational principle, the first staff member who encounters a request for mental health support is responsible for organizing the initial meeting. Finally, the last organizational principle, “psychological continuity,” refers to the idea that the staff members of the team become accountable for the treatment until its completion. Moreover, the different therapies that may be required (e.g., family, individual, group, occupational, and pharmacological) should be integrated into a continuous process.

These seven basic principles have been expanded and refined into 12 fidelity criteria that support the implementation of Dialogic Practice at the global level (Olson et al., 2014). The fidelity criteria were defined as follows: (1) two (or more) therapists in the team meeting; (2) participation of family and network; (3) use of open-ended questions; (4) respond to clients’ utterances; (5) emphasize the present moment; (6) eliciting multiple viewpoints; (7) use of a relational focus in the dialogue; (8) responding to problem discourse or behavior in a matter-of-fact style and attentive to meanings; (9) emphasizing the clients’ own words and stories, not symptoms; (10) conversation among professionals (reflections) in the treatment meetings; (11) being transparent; and (12) tolerating uncertainty.

These therapeutic elements are grounded in several key theoretical assumptions, which Seikkula and Olson (2003) define as the poetics of Open Dialogue.

At the core of the principle of tolerance of uncertainty is the idea that each crisis is unique and that maintaining a high tolerance for uncertainty in therapeutic work is essential. This principle encourages therapists to remain calm and avoid premature conclusions, even in high-risk and emotionally intense situations. By embracing the unknown and its inherent possibilities, new meanings can naturally emerge through collective dialogue. This approach is closely tied to the importance of establishing a trustworthy therapeutic context, where safety and trust are paramount for both therapists and families.

Dialogism, rooted in Bakhtin’s concept of dialogue (Bakhtin, 1984), views dialogue as both a process and an objective of therapy. During a crisis, it is essential to create an environment where all voices and perspectives are expressed and taken seriously. This approach transforms the experience of crisis from an isolating condition into a shared communicative process. The collaborative nature of this dialogue, supported by the presence of multiple facilitators, ensures that everyone feels heard and respected, which is crucial for building mutual understanding and trust within the network.

Polyphony involves recognizing and integrating multiple voices and perspectives into the therapeutic process. Open Dialogue shifts the focus from trying to modify fixed relational structures to fostering a dynamic, co-evolving dialogue where all participants can express their views. Reflective practice (Andersen, 1991) is essential for recognizing and utilizing this polyphony, enriching the therapeutic dialogue and promoting a deeper understanding of the crisis.

In addition to the core principles of Open Dialogue, several other values are central to this approach, namely equality, democracy, respect, transparency, and process orientation (Putman, 2021).

Open Dialogue emphasizes treating all voices equally in network meetings, ensuring that professional opinions do not dominate. This democratic approach reflects the Finnish cultural ethic, fostering respect for diverse and even conflicting viewpoints. When decision-making proves challenging, inviting additional perspectives can enhance the dialogue and provide fresh momentum.

Maintaining transparency, professionals avoid discussing the network without its members present, thereby reinforcing respect and strengthening the therapeutic process. Reflections are openly shared during meetings, allowing team members to address difficult topics skillfully and respectfully. This practice enriches understanding and supports the network’s ability to make sense of their experiences collectively.

The approach is inherently process-oriented rather than goal-oriented, emphasizing the experience of sustained participation in network meetings. Trusting the process involves the belief that ongoing engagement in these meetings will effectively address significant issues. This ongoing engagement gradually shifts communication from monologic to dialogic, fostering genuine dialogue, deeper understanding, and meaningful transformation in relationships and behaviors.

### 1.2 Research evidence and insights on Open Dialogue

Cohort studies investigating the OD approach in Western Lapland have demonstrated positive outcomes for almost 30 years (Seikkula et al., 2006, 2011; Bergström et al., 2018, 2022). The first cornerstone studies explored the effectiveness of OD within the Finnish national multicenter Integrated Treatment of Acute Psychosis (API) project (April 1992–December 1993) and its continuation, the Open Dialogue Approach in Acute Psychosis (ODAP) project (1994–1997; Seikkula et al., 2003, 2006). A third study, conducted between 2003 and 2005, examined whether previous results remained stable over the years (Seikkula et al., 2011).

Researchers have evaluated several outcomes in the treatment of first-episode psychosis, including psychotic symptoms, use of neuroleptic medications, number of relapses, employment status, and granting of disability allowance (Seikkula et al., 2011). They observed that in all three cohorts, more than 80% of patients had no residual symptoms at the two-year follow-up. Moreover, they found that only 16% of the patients in the ODAP2003-2005 group were on disability allowance, while 84% had returned to full employment or studies after 2 years of treatment.

A few years later, Bergström et al. (2018) compared a group of OD patients from the Western Lapland research cohort with a control group of patients who experienced first-episode psychosis and were referred to the Finnish public specialized healthcare system. The study confirmed that positive outcomes, such as the reduced need for psychiatric treatment or hospitalization and disability allowances, were maintained for over 19 years. Similarly, an evaluation of the treatment outcomes of a group of adolescents has recently highlighted how patients in the OD group were less likely to receive treatment or disability allowance at the 10-year follow-up (Bergström et al., 2022).

Although OD have been implemented in more than 20 countries (Pocobello et al., 2023), its transferability and positive outcomes have been demonstrated in very few contexts.

Gordon et al. (2016) explored the adaptation of Open Dialogue (OD) in the United States through the implementation of a program named the Collaborative Pathway (CP). The feasibility and effectiveness of CP were assessed using qualitative interviews, surveys, and clinical records. Despite the study’s limitations, such as a small sample size of only 14 patients, it yielded promising results concerning the transferability of the approach. Notably, network meetings generated high satisfaction levels among patients, their families, and staff members. Clinical outcomes, assessed through both surveys and clinical records, showed improvements in symptoms, functioning, and the need for care. Remarkably, more than half of the participants (nine out of 14) had returned to work or educational pursuits after 1 year of treatment.

Kinane et al. (2022) investigated the implementation and outcomes of a variation of Open Dialogue that incorporated peer support (POD), offered by a standalone team within the United Kingdom’s National Health System. This study employed a before-and-after design involving 50 service users and 25 carers over 6 months. Researchers assessed health and social function through both user self-reports and clinician-rated scales, as well as service experience, well-being, and carer support. All measures showed improvements from baseline scores at the three-and six-month marks, with an observed increase in employment or educational engagement.

Two other studies are currently underway to evaluate the efficacy of the OD approach. The first, conducted in the United Kingdom, is part of a comprehensive research initiative called ODDESSI (Open Dialogue: Development and Evaluation of a Social Network Intervention for Severe Mental Illness). This initiative includes the first randomized controlled trial of OD, with results expected this year (Pilling et al., 2022). Internationally, the HOPEnDialogue project^1^ seeks to synergize various global research efforts within the ODDESSI framework. Launched in June 2022, the project’s pilot phase is exploring the feasibility of conducting a multinational study and is assessing whether clinical outcomes associated with OD—such as time to relapse, quality of life, and social network dimensions—align with those observed in the ODDESSI trial (Pocobello, 2021).

Regarding qualitative studies, they have shown that clients and family members tend to value several dimensions of the OD approach, including network involvement, the shared decision-making process, and the sense of being heard (Tribe et al., 2019; Florence et al., 2021; Gidugu et al., 2021; Buus and McCloughen, 2022). Similar experiences have also been observed in the long term, as shown in a study of service users from the original Western Lapland research cohort, who were interviewed 10–23 years after their first OD treatment (Bergström et al., 2022). Participants indicated that they appreciated attending network meetings, the interest shown by other people, and the opportunity to discuss their experiences openly and without feeling judged. On the other hand, mixed feelings were reported about some features of the OD approach, such as the immediate response (i.e., staff arriving suddenly at the client’s home), teamwork (i.e., too many people attending meetings), hospitalization, and medication (Bergström et al., 2022).

In different implementation contexts, clinicians’ experiences of OD have been associated with both opportunities and challenges (Florence et al., 2020; Dawson et al., 2021; Schubert et al., 2021; Jacobsen et al., 2023; Skourteli et al., 2023). Professionals participating in network meetings reported positive feelings such as a sense of liberation, collaboration, humanity, authenticity, and identity change (Florence et al., 2020; Dawson et al., 2021; Schubert et al., 2021; Jacobsen et al., 2023). Difficulties included, for example, that some practitioners felt burdened with responsibility, especially when unit managers were not supportive and engaged in the development of the approach (Jacobsen et al., 2023). Others found it difficult to link theory to practice, particularly in relation to transparency and reflective practice, and to manage uncertainty by giving up the need for control (Skourteli et al., 2023). Psychiatrists reported discomfort in dealing with situations of perceived high risk, describing vulnerability as “the greatest strength and the greatest challenge” (Schubert et al., 2021). Further research is needed to describe these barriers in different contexts and to help overcome them as the approach is implemented in clinical practice.

### 1.3 Open Dialogue in the Italian context

The research described in this paper was partially conducted under a project funded by the Italian Ministry of Health (Program CCM 2014), aimed at evaluating the transferability of the OD approach within the Italian National Health Service, which manages mental health care at the community level through Mental Health Departments (MHDs). Each MHD comprises all services and facilities devoted to mental health care, assistance, and prevention for users within a defined catchment area (Lora, 2009). MHDs may include Community Mental Health Centers (CMHCs; Centri di Salute Mentale), Day Care Facilities (DCF; Centri Diurni), General Hospital Psychiatric Units (GHPUs), and Residential Facilities (RFs).

The Open Dialogue (OD) project, initiated in February 2015, involved eight Mental Health Departments (MHDs) across six Italian cities—Catania, Modena, Rome, Savona, Trieste, and Turin—serving a population of 4 million inhabitants. Importantly, OD was not implemented across entire departments but was selectively applied in specific areas, chosen based on team size and the organizational structure of each department.

The participating centers were invited by the coordination unit to join the project based on their interest and curiosity in learning about the Open Dialogue approach, as well as their expertise in similar collaborative approaches. Many professionals within these centers were already in contact with each other, sharing a common interest in recovery-based services, voice hearers’ groups, democratic communities, and multi-family groups. Each department then selected candidates for training from those who volunteered. The entire process was based on motivated, committed, and voluntary participation at all levels.

Initially conceived as a two-year project, this initiative comprised 3 months of training followed by a year-long outcome study. It soon became apparent that a minimum of 1 year was essential to provide comprehensive foundational training in OD. This necessary extension delayed the initiation of the outcome study. Despite a brief extension granted toward the funding period’s conclusion, the outcome study began with limited time remaining and proceeded without additional financial support. Subsequently, one department ceased participation following the formal conclusion of the project and did not continue into the outcome study phase.

The Italian OD project encompassed training and supervision for mental health professionals and explored the transferability of the approach through a structured research program. The Local Health Authority of Turin coordinated the project, while the National Research Council (CNR) oversaw the evaluation process. The program was divided into several phases: preliminary assessment, training, and an outcome study, each linked to a specific research focus.

In the preliminary assessment phase, the CNR unit conducted detailed evaluations through interviews with directors of the MHDs and questionnaires distributed to health professionals. This phase aimed to gauge the compatibility of the OD practices with the values and needs of both professionals and their organizations, identifying potential barriers and formulating strategies for implementation.

The training program engaged 80 mental health professionals, including psychiatrists, nurses, psychotherapists, social workers, and one expert by experience, who were organized into two classes. Initially, participants completed sessions on family therapy led by Italian psychotherapists. This was followed by 20 days of intensive OD training delivered by Finnish trainers. Supervision, a crucial aspect of the training, extended slightly beyond the planned year. The training phase was closely monitored through participatory observation by the evaluation unit.

To evaluate the adherence of professionals to OD principles during network meetings, each team submitted two video recordings at the training’s conclusion. These recordings were analyzed using the Dialogic Practice Adherence Scale (Olson et al., 2014) by independent raters. The analysis confirmed sufficient adherence to OD practices (Ciliberto et al., 2017; Pocobello and el Sehity, 2017; Pocobello, 2021), which was vital for ensuring the professionals’ practices met the rigorous standards required for faithful implementation of the OD approach. Only after achieving satisfactory fidelity and adherence scores did we move to the next phase.

The start date of the outcome study varied among the different MHDs in relation to the timing of approval from the local ethical committees; however, in all departments, the research concluded before November 2018. This final phase applied the skills and principles from the training in practical settings to evaluate the clinical outcomes and overall effectiveness of the OD approach in the Italian context.

Results from all phases were systematically reviewed in project coordination meetings, which facilitated informed decisions and adjustments throughout the implementation process. This structured approach ensured that each phase built upon the insights gained from the previous, enhancing the integrity and impact of the research presented in this article.

### 1.4 Aims

This study aims to quantitatively document both the implementation and the outcomes of the OD approach within Italian MHDs. The objectives include:

- Evaluating how patients and their families perceive OD network meetings.
- Analyzing the clinical outcomes for patients over a 12-month period.
- Assessing perceived changes in the social networks.

## 2 Methods

### 2.1 Study design

The study is a 12-month multisite prospective cohort study. Patients aged 18–64 years were included. Measurements were taken at baseline (t1), after 6 months (t2), and after 12 months (t3). Outcome variables are the Global Assessment of Functioning (GAF), Clinical Outcomes in Routine Evaluation—Outcome Measure (CORE-OM), and the Lubben Social Network Scale (LSNS-6). OD-Sessions were rated via two scales: Session Rating Scale (SRS) and Outcomes Rating Scale (ORS).

### 2.2 Sampling and recruitment process

For 1 month, the teams practiced OD to treat all individuals aged 18–64 who were seeking help for the first time in the designated area, continuing until their capacity to manage additional new requests according to OD principles was reached (Olson et al., 2014). No distinctions were made based on diagnosis, and all types of initial crises and requests for help were addressed using OD.

### 2.3 Data collection procedures

Immediately upon the initial call for help, patients were contacted within 24 h for treatment at their preferred location. A team committed to ensuring continuity of care throughout the treatment duration was assigned. At the first or second meeting with the patient (t0 = baseline), the opportunity to participate in the research was presented, and informed consent was obtained.

Following consent, data collection began, which included socio-demographic details and clinical diagnostics according to ICD-10. The scales utilized for further assessments were the CORE-OM (Evans et al., 2002) for monitoring routine clinical outcomes, the GAF (Endicott et al., 1976) to evaluate overall functioning, and the LSNS-6 (Lubben et al., 2006) to measure the size of the patient’s social network. These measures were taken at baseline and subsequently at 6 and 12 months to track the effectiveness of the clinical interventions.

For process documentation and evaluation, the Session Rating Scale (Duncan et al., 2003) was used after each meeting to gauge satisfaction with the care received by patients and their networks. Additionally, the Outcome Rating Scale (Miller et al., 2003) was administered every 2 weeks during scheduled meetings to continuously assess perceived outcomes.

### 2.4 Measurement tools and variables

#### 2.4.1 Instruments for process evaluation

##### 2.4.1.1 SRS

The Session Rating Scale (Duncan et al., 2003) is a client-reported outcome measure designed to evaluate the therapeutic alliance and session satisfaction in individual network meetings. It consists of a single item in which clients rate their overall experience of the session on a 0–10 scale. SRS allows clients to provide feedback on various aspects of the therapeutic process, including the quality of the therapeutic relationship, the perceived helpfulness of the session, and their level of engagement. It serves as a simple yet valuable tool for therapists to monitor and assess a client’s experience, identify areas of improvement, and enhance the effectiveness of therapy by incorporating client feedback into the treatment process.

##### 2.4.1.2 ORS

The Outcome Rating Scale (Miller et al., 2003) is a client-reported outcome measure used to assess the overall outcome and progress of therapy. It consists of four items that cover different domains of well-being: individual well-being, interpersonal relationships, social roles, and overall satisfaction with life. Clients rated their level of functioning in each domain on a 0–10 scale, providing a snapshot of their subjective experience and perceived improvement over time. The ORS is a valuable tool for monitoring treatment progress, evaluating therapeutic outcomes, and facilitating client-centered discussions regarding goals and areas of focus in therapy. This enables therapists to incorporate client feedback, track changes, and tailor interventions to address specific needs and concerns.

#### 2.4.2 Instruments for the evaluation of outcome

##### 2.4.2.1 GAF

The Global Assessment of Functioning (Endicott et al., 1976) scale is a clinician-rated measure that assesses an individual’s overall level of psychological, social, and occupational functioning. It is commonly used in mental health settings to evaluate functional impairment and overall wellbeing. The GAF scale rates individuals on a continuum from 0 to 100, with lower scores indicating greater impairment, and higher scores indicating better functioning. It considers various factors, such as symptoms, functioning in daily life, social relationships, and work/school performance. The GAF scale provides a summary score that helps clinicians gauge the severity of mental health conditions, track changes over time, and inform treatment plans and interventions. The scale is widely used in routine clinical settings (Aas, 2010).

##### 2.4.2.2 CORE-OM

The Clinical Outcomes in Routine Evaluation-Outcome Measure (Evans et al., 2002) is a self-report questionnaire designed to assess psychological distress and well-being among individuals receiving mental health services. It consists of 34 items covering four domains: subjective well-being, symptoms/problems, functioning, and risk/harm. Respondents rated each item on a five-point Likert scale indicating the extent to which they experienced specific difficulties or distress over the past week. The CORE-OM scale provides a comprehensive assessment of a person’s emotional and psychological states, allowing clinicians and researchers to monitor treatment progress, evaluate outcomes, and identify areas of concern in mental health interventions. The scale had a high level of internal consistency across the three different time points (t0; t1; t2) as determined by Cronbach’s alphas of 0.937; 0.951; 0.949, respectively.

##### 2.4.2.3 LSNS-6

The Lubben Social Network Scale-6 (Lubben et al., 2006) is a brief self-report questionnaire used to assess social isolation and support among older adults. It consists of six items that capture both the structural aspects of social networks (e.g., frequency of contact and number of close relationships) and the functional aspects of social support (e.g., availability of emotional support and practical assistance). The LSNS-6 scale provides a quick and reliable measure of an individual’s social connectedness and can help identify older adults who may be at risk of social isolation or lack sufficient social support. The LSNS-6 was employed in this study to assess social networks and social support, and to screen for the social isolation of patients. The scale is constructed from two sets of three questions: one forming the family subscale and the other forming the friends’ subscale. The scale had a high level of internal consistency across the three time points (t0; t1; t2) as determined by Cronbach’s alpha of 0.84; 0.80; 0.84, respectively, similar to the consistency described by Lubben et al. (2006) of 0.83.

### 2.5 Sample

During the one-month recruitment phase, 125 individuals reached out for assistance within the designated catchment areas. Of these, 21 were deemed ineligible for the study for the following reasons: 9 due to their sole request of medical certifications, 7 because they were not first-time patients, 3 fell outside the age criteria of the study, and 2 due to their sole requested of a physician. This resulted in 104 potentially eligible participants of whom 32 chose not to participate; their reasons included reluctance of their social network to participate (14 cases), refusal to be part of a study (12 cases), and discomfort speaking in front of multiple people (6 cases). Thus, 72 participants were eligible yielding a recruitment rate of 69.2%. Due to the withdrawal of one mental health department after the first moth of the study the data of 8 participants were lost; 6 more participants disengaged after the first month of the study, bringing about an attrition rate of 19.4%. Of the remaining 58 participants, data were missing for 11 users at month 6 and for 11 users at month 12. In total, 40 users had complete data at all three time points. Details of the participant characteristics are summarized in Table 1.

**Table 1:** Characteristics of study participants at baseline, 6 months, and 12 months.

Characteristics	Baseline (*N* = 58)	Month 6 (*N* = 47^*^)	Month 12 (*N* = 47^*^)
**Sociodemographic**			
Men, *n* (%)	21 (36.2)	15 (31.9)	17 (36.2)
Women, *n* (%)	37 (63.8)	32 (68.1)	30 (63.8)
*Age* at baseline, *M (SD)*	36.4 (13.9)	37.9 (14.4)	36.8 (14.3)
***Studies* at baseline (missing, *n* = 1)**			
Studies, *n* (%)	12 (20.7)	9 (19.1)	10 (21.3)
No Studies, *n* (%)	45 (77.6)	38 (80.9)	37 (78.7)
***Occupational Status* at baseline (missing, *n* = 2)**			
Work, *n* (%)	31 (53.4)	28 (59.6)	27 (57.4)
No Work, *n* (%)	25 (43.1)	18 (38.3)	20 (42.6)
***Relationship status* at baseline**			
Married/Cohabits, *n* (%)	14 (25.0)	14 (29.8)	12 (25.5)
Divorced/Separated, *n* (%)	7 (12.5)	4 (8.5)	4 (8.5)
Single/Widowed, *n* (%)	37 (62.5)	29 (61.7)	31 (66.0)
**Clinical characteristics**			
***ICD 10 Diagnostic* (missing, *n* = 12)**			
F10–F19 Mental and behavioral disorders due to psychoactive substance use, *n* (%)	1 (2.2)	0 (0.0)	0 (0.0)
F20–F29 Schizophrenia, schizotypal and delusional disorders, *n* (%)	8 (19.6)	8 (21.6)	8 (21.6)
F30–F39 Mood [affective] disorders, *n* (%)	10 (21.7)	6 (16.2)	7 (18.9)
F40–F48 Neurotic, stress-related and somatoform disorders, *n* (%)	20 (43.5)	17 (45.9)	17 (45.9)
F50–F59 Behavioral syndromes associated with physiological disturbances and physical factors, *n* (%)	1 (2.2)	1 (2.7)	1 (2.7)
F60–F69 Disorders of adult personality and behavior, *n* (%)	4 (6.9)	3 (8.1)	4 (10.8)
F70–F79 Mental retardation, *n* (%)	2 (3.4)	2 (5.4)	0 (0.0)

^*^The participants at time points month 6 and month 12 are not identical since of 18 participants only 2 two time-point measures were available.

### 2.6 Data analysis strategies

Firstly, descriptive statistics are provided for sample characteristics. An analysis of variance (ANOVA) was conducted to examine the age differences of the session participants based on their roles and gender.

We carried out an analysis to examine patterns of missing values in our process (SRS and ORS) and outcome variables (GAF, CORE-OM, LSNS-6) to determine if the data were missing at random. This step was crucial for validating the assumptions of our mixed model analysis. The results confirmed that incomplete data were indeed distributed at random. We then employed a linear mixed-effects model to analyze the longitudinal data collected across multiple time points. This statistical approach was chosen due to its ability to reduce the loss of information about patients of which data of only two timepoints were available (Heck et al., 2022). Linear mixed models use maximum likelihood estimation, which allows them to incorporate all available information even when there are missing data points, which is in contrast to repeated-measures ANOVA, which typically removes incomplete cases (de Melo et al., 2022). Each subject’s repeated observations were modeled with fixed effects for time, capturing the systematic changes in the dependent variable, while random intercepts were included to account for individual differences at baseline. The models were fitted using Restricted Maximum Likelihood (REML) estimation to provide unbiased estimates of the variance components under the assumption that the fixed effects are correctly specified. This modeling strategy allowed us to directly assess the impact of time on the outcome measure while controlling for within-subject correlation and between-subject heterogeneity. Model fit was evaluated using the Akaike Information Criterion (AIC) and Bayesian Information Criterion (BIC), and the proportion of variance explained by the models was quantified using marginal and conditional R-squared values. Residual diagnostics were performed to assess assumptions of normality and homoscedasticity, ensuring the robustness of our inferences.

Linear mixed models were calculated using Jamovi (The Jamovi Project, 2022) module for General analyses for linear models (Gallucci, 2019).

## 3 Results

### 3.1 Descriptives of OD-network meetings

517 OD network meetings with 58 patients were reported during the 12 months duration of the study. The average number of OD-network meetings per patient treated was 8.08 (*SD* = 5.74; *Md* = 6; *Min* = 1; *Max* = 25) and an average number of social network members participating in OD-network meetings was 0.96 (*SD* = 0.90; *Md* = 1; *Min* = 0; *Max* = 7). The number of social network members participating in OD-network meetings was 17% higher for male patients than for female patients (*B* = 0.167; *SE* = 0.06; *p* = 0.006).

Of the 517 OD-network meetings 158 meetings (30.6%) included only the patient, 217 (42%) meetings included one social network member, 98 meetings (19%) included two members, 30 (5.5%) three members and 14 (2.7%) meetings included four or more members (max. 8).

**Figure 1:**
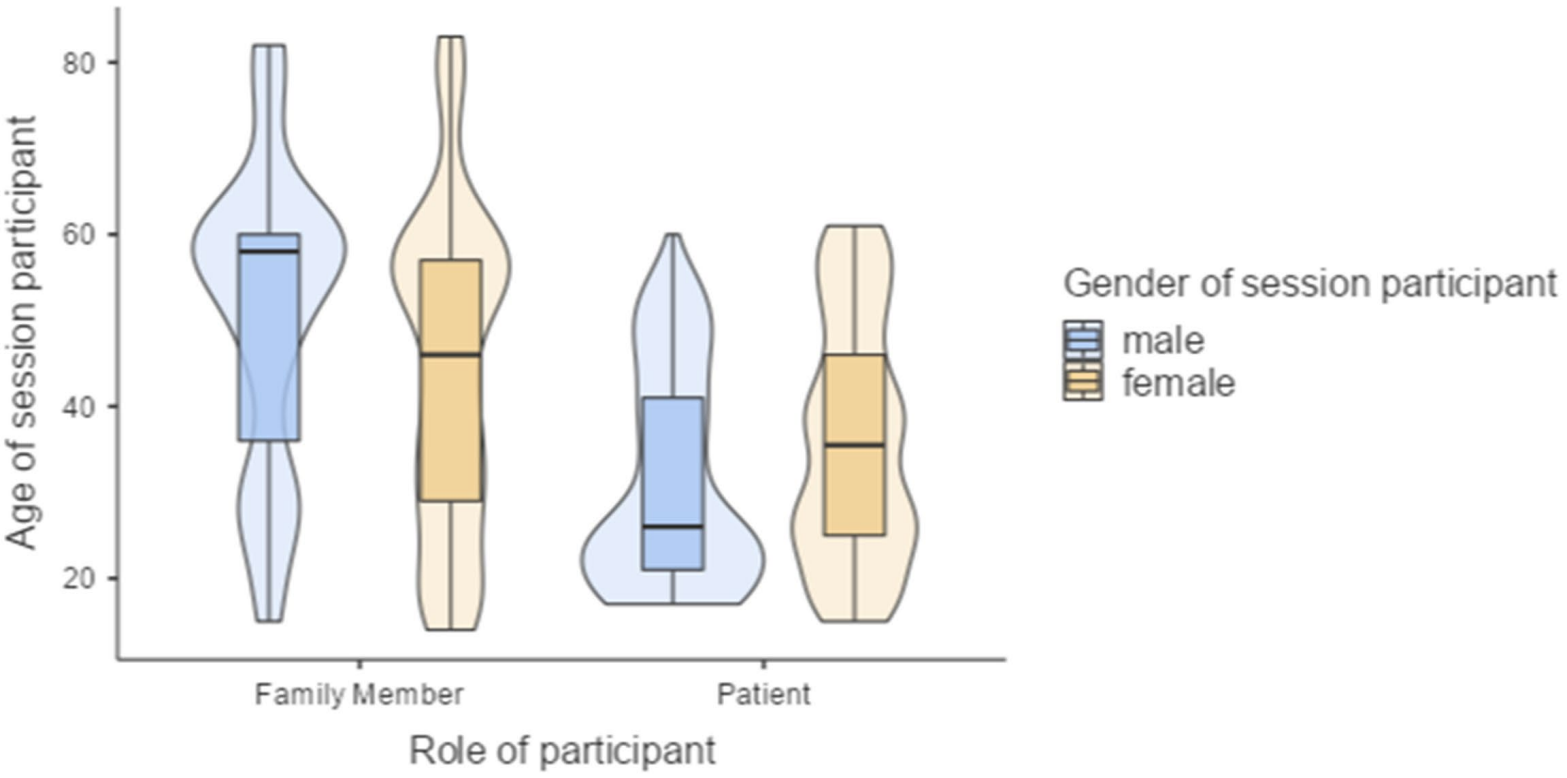
Age structure and gender of OD-network meeting participants based on their role as social network members and patients.

28% of the network meetings were rated by patients’ mothers, 18% fathers, 14% partners, 10% sisters, 8% brothers, 2% daughters and 2% others. Consequently, the age structure between genders varied systematically based on their role in OD-session as patients or social network members. The mean age of social network members was 46.4 years (*SD* = 18.0; min. 15.0 to max. 82.0); the mean age of patients was 36.4 years (*SD* = 13.4), ranging from 18.0 to 61.0 years (see Table 1). The results of a one-way ANOVA revealed a significant main effect of the role of participants on age [*F*(1, 123) = 11.62, *p* < 0.001], indicating that social network members tended to be older than patients. However, there was no significant main effect of gender of session-participants on age [*F*(1, 123) = 0.08, *p* = 0.780]. Furthermore, *post hoc* comparisons revealed that there were no significant age differences between male patients and female patients (*p* = 0.357; see Figure 1).

#### 3.1.1 Session rating scale of OD-network meetings

1,080 session rating scales (SRS) were completed to assess 517 OD-network meetings. 517 SRS were completed by patients (*M* = 34.9; *SD* = 7.17; *Md* = 38.8;) and 563 SRS by members of their social network (*M* = 34.4; *SD* = 6.79; *Md* = 36). A one-sample t-test revealed that these SRS scores were significantly above the mean global SRS-scores of 32.4 (*SD* = 5.9; *t* = 7.905, *p* < 0.001) reported in the cross-cultural examination of the scale by Hafkenscheid et al. (2010). The data were skewed to the higher endo of the scale indicating the prevalence of positive ratings of the OD-network meetings.

To explore patterns in the appreciation of OD-network meetings throughout the therapeutic journey, the rank-order of OD-network meetings was standardized: (1st, 2nd, 3rd …) divided by the total number of OD-network meetings recorded so that the last OD-session was designated with the reference value 1 and all earlier OD-network meetings were allocated a “temporal order” score approximating 0; (2) the role of session participants (patient vs. social network member). The linear mixed model analysis examined the association between the SRS and the following predictors: (1) Role of session participant, (2) standardized rank-order of OD-network meetings (ranging from <0 to 1, where 1 represents the last session), and (3) the interaction between Role of session participant and standardized rank-order of OD-session. The model included random intercepts for social network and individual level. The fixed effects omnibus tests indicated a marginally significant effect for role of session participant (*F* = 3.38, *p* = 0.066) and a significant effect for Order of OD-session (*F* = 4.07, *p* = 0.044), suggesting that these variables were associated with the SRS scores. There was no statistically significant interaction effect between role of session participant (patient vs. social network members) and temporal rank order of OD-session (*F* = 1.95, *p* = 0.163).

**Figure 2:**
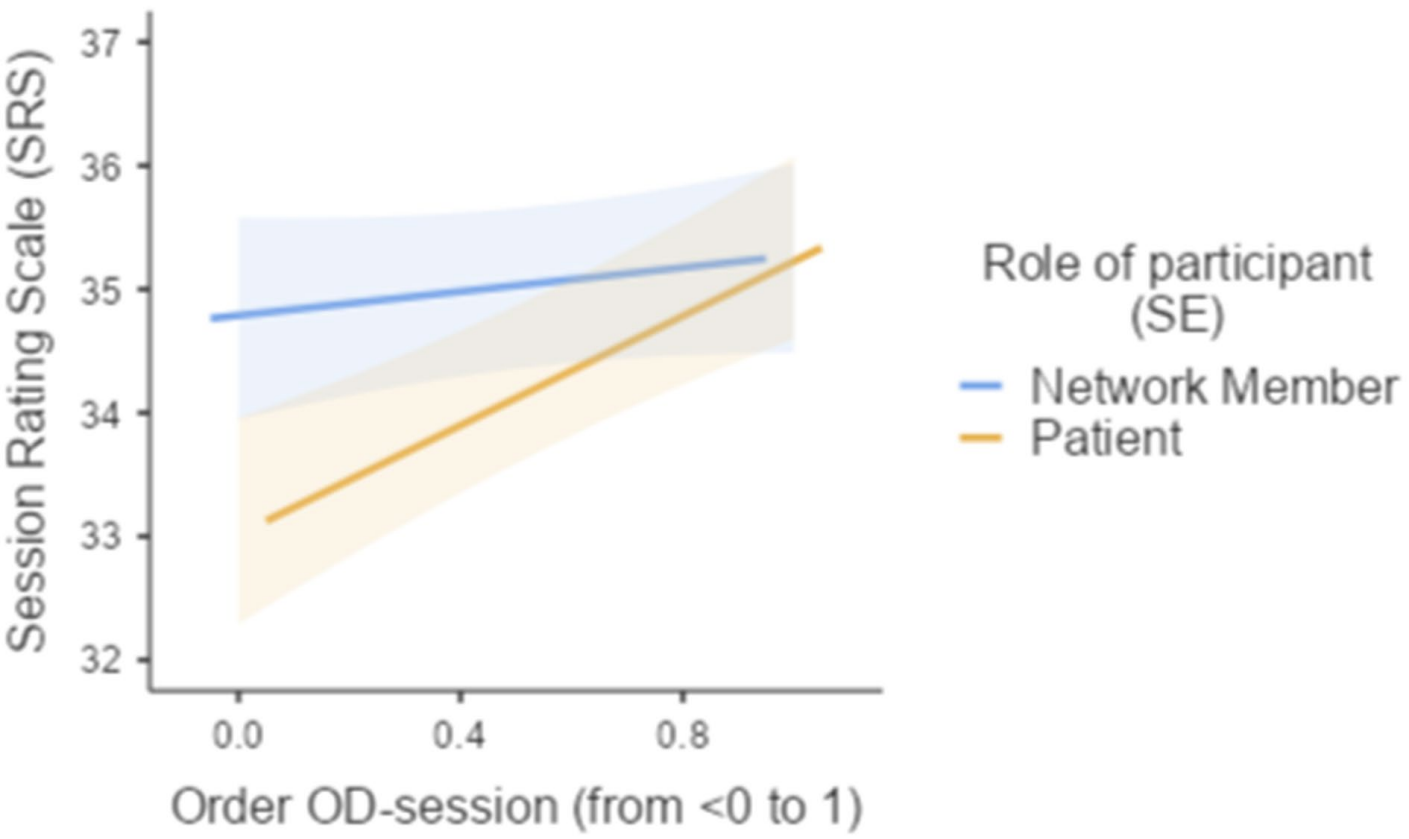
Effects plot of SRS and the order of OD-network meetings during the OD-therapy.

The fixed effects parameter estimates showed that there was no significant difference in SRS-scores between patients and their social network members (*B* = −0.693, *SE* = 0.377, *p* = 0.066). Overall, the interaction between role of session participants (patient or social network member) and the rank of OD-session did not significantly influence SRS scores (*B* = 1.727, *SE* = 1.235, *p* = 0.163). Patients, however, rated later OD-session in the therapy significantly more positively than their early OD-network meetings (*B* = 1.347, *SE* = 0.668, *p* = 0.044; see Figure 2).

#### 3.1.2 Outcome rating scale of OD-network meetings

**Figure 3:**
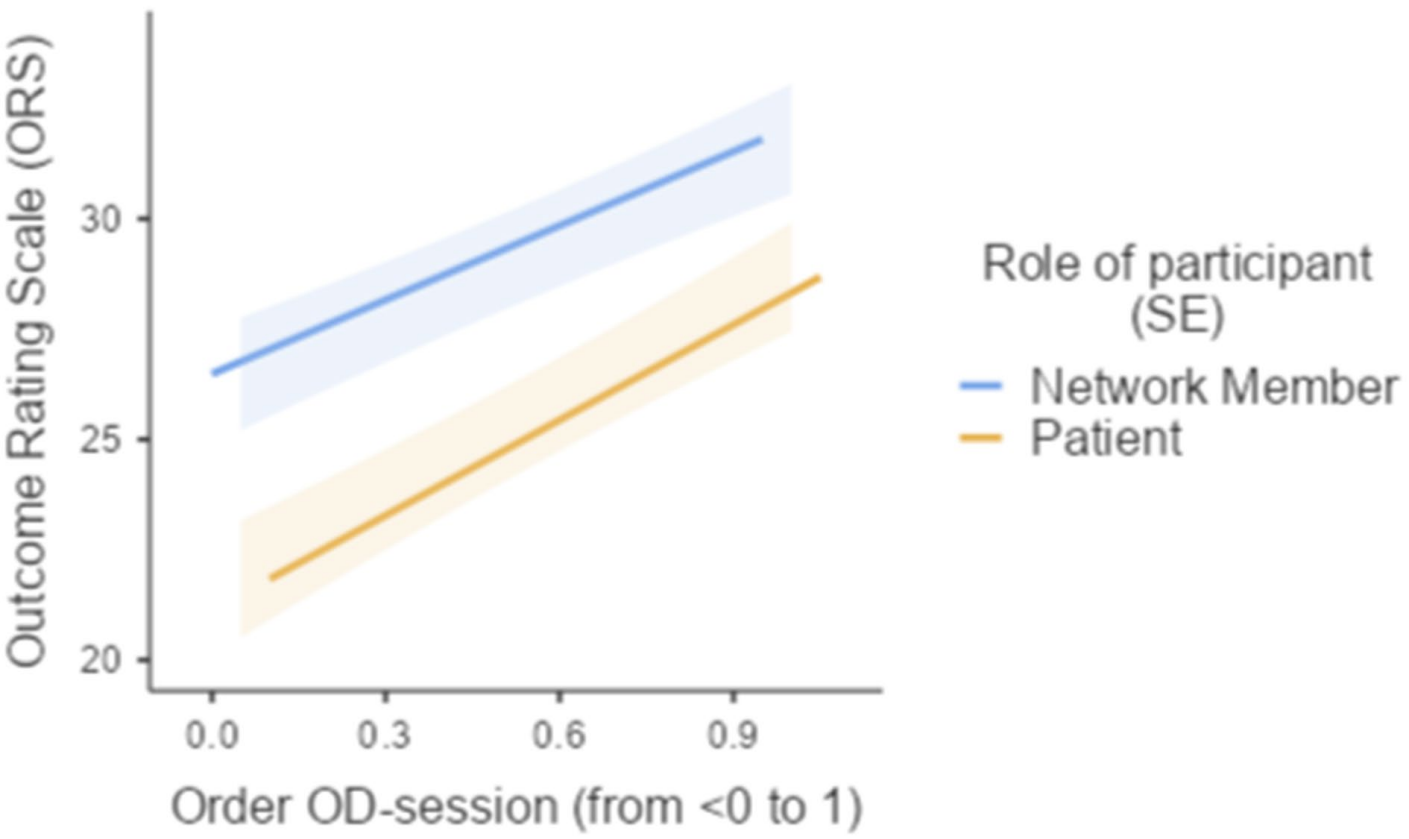
Effects plot of ORS and the order of OD-network meetings during the OD-therapy.

A mixed model analysis was employed to explored the relationship between the Outcome Rating Scale (ORS) and the following predictors: Role of session participant (Patient or Social network members), standardized rank-order of OD-network meetings, and the interaction between role of session participant and standardized rank-order of OD-session. The model included random intercepts for Social network and individual level ratings (“Super ID”). The fixed effects parameter estimates indicated that patients rated the outcome of OD-network meetings significantly lower than their social network members (*B* = −4.73, *SE* = 1.26, *p* < 0.001), while the Order of OD-session was positively associated with ORS scores (*B* = 6.40, *SE* = 1.01, *p* < 0.001). The interaction between Role of session participant and Order of OD-session did not have a significant effect on ORS scores (*B* = 1.60, *SE* = 1.99, *p* = 0.421; see Figure 3).

In conclusion, the multilevel mixed model analysis showed that the role of session participant and order of OD-session were significant predictors of ORS scores. Patients rated the outcome of OD-network meetings lower compared to social network members, and the outcome of OD-session was rated higher over the course of the OD-therapy.

### 3.2 Outcomes evaluation

Table 2 presents longitudinal data on clinical outcomes measured across three time points: baseline, 6 months, and 12 months. The outcomes include the Global Assessment of Functioning (GAF), various dimensions of the Clinical Outcomes in Routine Evaluation—Outcome Measure (CORE-OM), and scores from the Lubben Social Network Scale (LSNS). This table provides a comprehensive overview of changes in mental health and social support over the course of the study, reflecting both individual and aggregate trends in the participant sample.

**Table 2:** Global assessment of functioning (GAF), CORE-OM, Lubben social network scale (LSNS).

Clinical outcomes	Baseline (*N* = 58)	Month 6 (*N* = 47)	Month 12 (*N* = 47)
**Global Assessment of Functioning (GAF)**			
*M (SD); n*(t)	63.3 (13.85); 56	70.81 (11.73); 47	77.4 (14.3); 47
**CORE-OM (** Evans et al., 2002 **)**			
Well-being, *M (SD)*	2.42 (0.94)	1.71 (1.01)	1.77 (1.06)
Symptoms, *M (SD)*	2.09 (1.00)	1.43 (0.87)	1.50 (0.93)
Functioning, *M (SD)*	1.70 (0.75)	1.35 (0.71)	1.50 (0.77)
Risk, *M (SD)*	0.63 (0.74)	0.30 (0.53)	0.33 (0.53)
CORE-OM no R, *M (SD)*	1.97 (0.78)	1.43 (0.76)	1.54 (0.82)
CORE-OM, *M (SD); n*(t)	1.73 (0.73); 56	1.23 (0.70); 47	1.33 (0.74); 42
**Lubben social network scale (LSNS-6)**			
Family Subscale, *M (SD)*	2.24 (1.03)	2.49 (1.05)	2.33 (0.95)
Friends Subscale, *M (SD)*	2.27 (1.10)	2.53 (1.20)	2.49 (1.17)
LSNS-6, *M (SD); n*(t)	2.26 (0.95); 57	2.51 (0.93); 48	2.41 (0.94); 42

#### 3.2.1 GAF scores: a linear mixed model analysis

A linear mixed-effects model was fitted using Restricted Maximum Likelihood (REML) to investigate the influence of time on the General Assessment of Functioning (GAF) scores, accounting for random intercepts for individual subjects (RID). Tests for normality of residuals indicated that the residuals were approximately normally distributed, as shown by the Kolmogorov–Smirnov test (D = 0.0601, *p* = 0.650) and the Shapiro–Wilk test (W = 0.9853, *p* = 0.112). The model used the formula GAF~1 + time + (1 | RID). The analysis yielded an Akaike Information Criterion (AIC) of 1166.373 and a Bayesian Information Criterion (BIC) of 1174.169. The model’s marginal R-squared was 0.155, suggesting that fixed effects alone accounted for approximately 15.5% of the variance in GAF scores, while the conditional R-squared was 0.613, indicating that the total model, including random effects, explained 61.3% of the variance.

The model included random intercepts for RID, which demonstrated a standard deviation of 9.67, corresponding to a variance of 93.6. The intraclass correlation coefficient (ICC) was 0.542, indicating that approximately 54.2% of the variability in GAF scores was due to differences between subjects.

**Figure 4:**
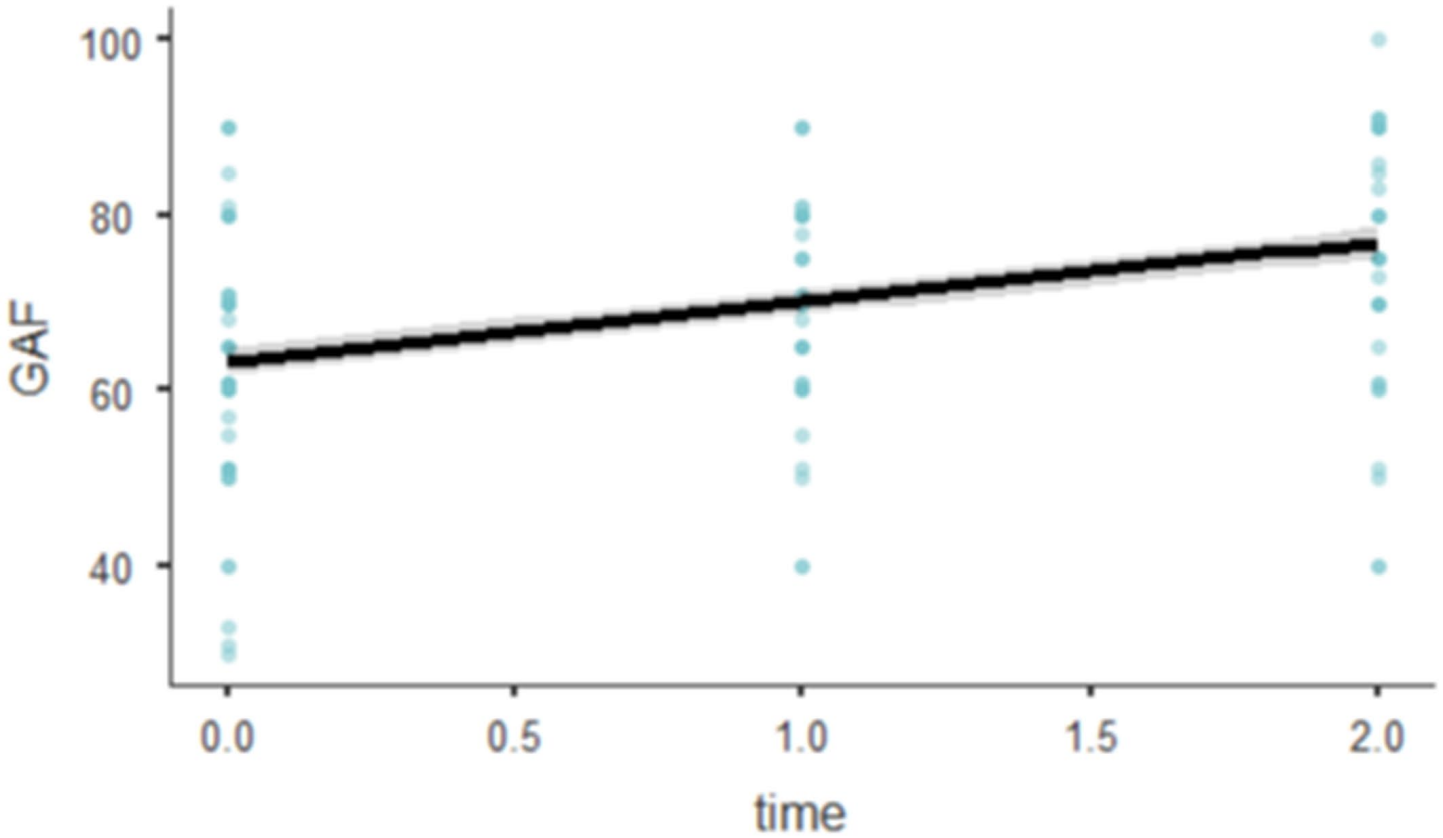
Effects plots of GAF.

The effect of time on GAF was statistically significant, with an F-statistic of 56.7 (df = 1, 101, *p* < 0.001), indicating a substantial effect over time. Specifically, the GAF scores increased by 6.78 for each additional time unit (SE = 0.901, 95% CI [5.02, 8.55], t(100.9) = 7.53, *p* < 0.001; Figure 4).

#### 3.2.2 CORE-OM

A linear mixed-effects model was applied to evaluate the influence of time on CORE-OM scores, accounting for random intercepts for individuals (RID). Tests for the normality of residuals indicated no violations of normality: Kolmogorov–Smirnov test (D = 0.0532, *p* = 0.806) and Shapiro–Wilk test (W = 0.9861, *p* = 0.152), suggesting that the assumption of normally distributed residuals holds for this model. The model was fitted using Restricted Maximum Likelihood (REML). The analysis resulted in an Akaike Information Criterion (AIC) of 287.5776 and a Bayesian Information Criterion (BIC) of 306.7380. The marginal R-squared was 0.0612, suggesting that fixed effects explained approximately 6.12% of the variance in CORE scores. The conditional R-squared was substantially higher at 0.5917, indicating that including random effects accounts for approximately 59.17% of the variance.

**Figure 5:**
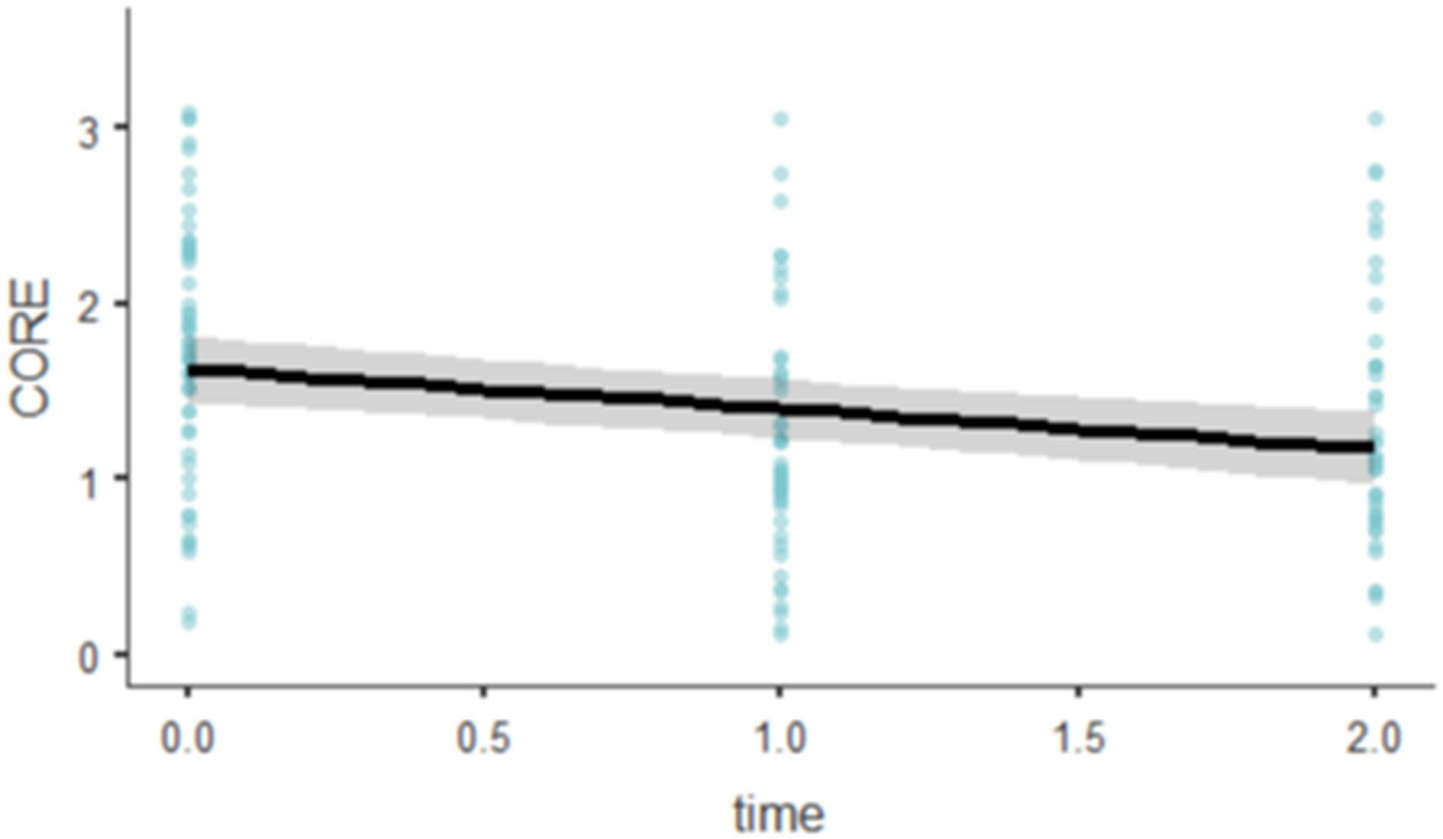
Effect plots of CORE-OM.

The fixed effect of time on CORE scores was significant, *F*(1, 94.1) = 19.8, *p* < 0.001. The model estimated a significant decrease in CORE scores over time, with each unit increase in time associated with a decrease of 0.228 in CORE scores (SE = 0.0511, 95% CI [−0.328, −0.127], t(94.1) = −4.45, *p* < 0.001; Figure 5).

#### 3.2.3 Lubben Social Network Scale

A linear mixed-effects model was conducted to assess the effect of time on Lubben Social Network Scale (LSNS) scores, accounting for random intercepts associated with individual subjects (RID). Tests for the normality of residuals revealed a deviation from normality with the Shapiro–Wilk test (W = 0.9681, *p* = 0.002), suggesting potential issues with the normal distribution assumption of the residuals, although the Kolmogorov–Smirnov test did not show significant results (D = 0.0952, *p* = 0.139). The model was fitted using Restricted Maximum Likelihood (REML). It provided an Akaike Information Criterion (AIC) of 345.2901 and a Bayesian Information Criterion (BIC) of 363.7063. The analysis showed a marginal R-squared of 0.0101, indicating that the fixed effects explained approximately 1.01% of the variance in LSNS scores. The conditional R-squared was significantly higher at 0.6683, suggesting that including random effects accounts for about 66.83% of the variance.

The random effects indicated a standard deviation of 0.764 for the intercepts across RID, corresponding to a variance of 0.584. The intraclass correlation coefficient (ICC) was 0.665, reflecting a substantial portion of the variability in LSNS scores attributable to differences among subjects.

**Figure 6:**
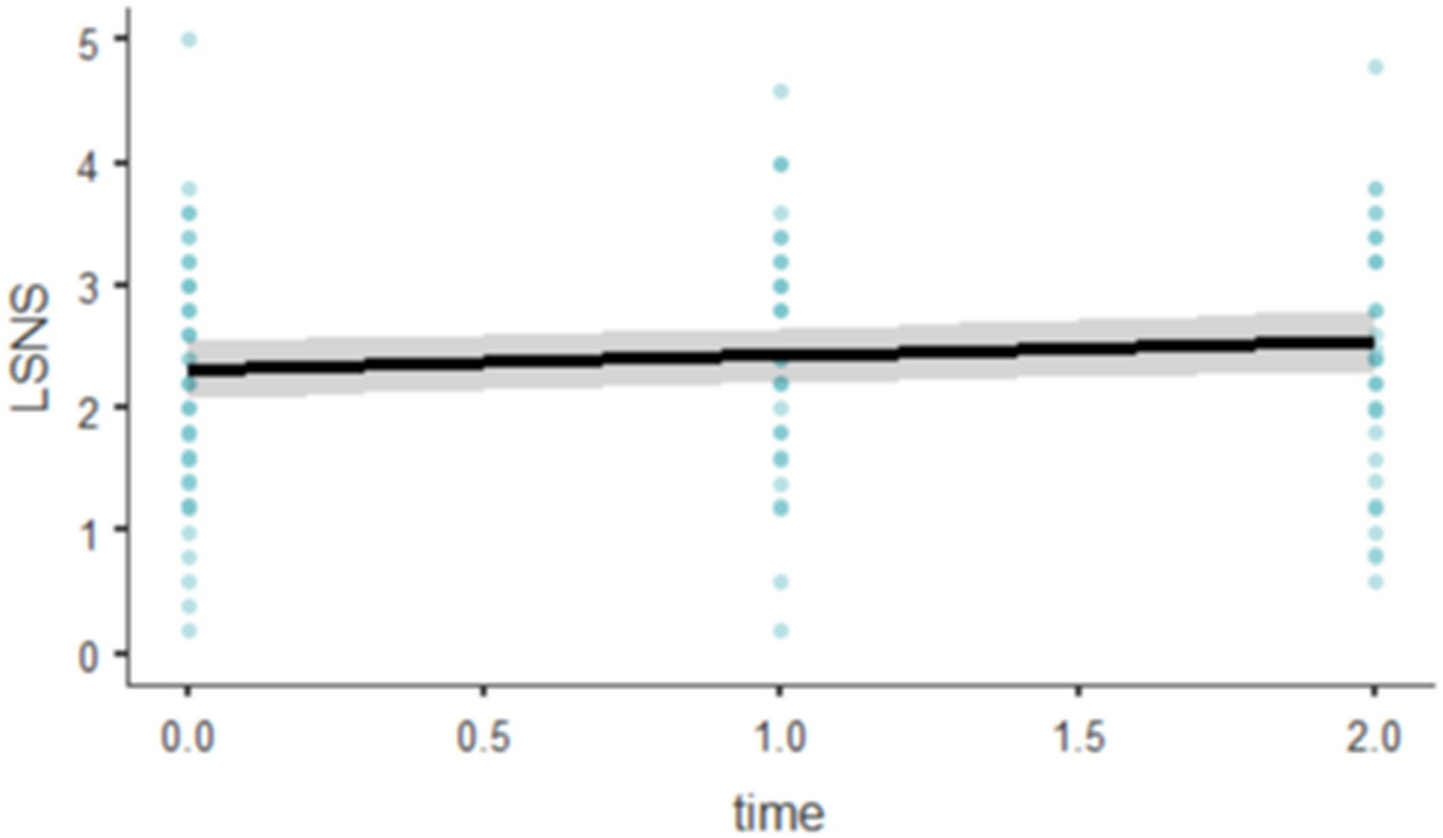
Effect plots of LSNS-6.

The fixed effect of time was statistically significant, *F*(1, 93.4) = 4.06, *p* = 0.047. The parameter estimate for time indicated a positive effect, with each unit increase in time associated with an average increase of 0.116 in LSNS scores (SE = 0.0574, 95% CI [0.00321, 0.228], t(93.4) = 2.02, *p* = 0.047; Figure 6).

Table 3 consolidates the key model parameters and fit statistics derived from the linear mixed models for each of the three outcome variables—Global Assessment of Functioning (GAF), Clinical Outcomes in Routine Evaluation (CORE-OM), and the Lubben Social Network Scale (LSNS). This table provides a summary of the estimates, standard errors, degrees of freedom, t-values, *p*-values, and confidence intervals for both intercepts and time effects across the models. Additionally, the table displays the marginal and conditional R^2^ values, which help quantify the proportion of variance explained by the fixed effects alone and by the entire model respectively, offering insights into the effectiveness of the interventions over time.

**Table 3:** Model parameters and fit statistics for linear mixed model of the three outcomes.

Outcome	Parameter	Estimate	SE df	Df	t	*p*-value	95% CIT	Marginal R2	Conditional R2
GAF	Intercept	69.81	1.489	58.7	46.90	<0.001	[66.89, 72.72]	0.155	0.613
	Time	6.78	0.901	100.9	7.53	<0.001	[5.02, 8.55]		
CORE-OM	Intercept	1.629	0.094	87.5	17.35	<0.001	[1.445, 1.813]	0.061	0.592
	Time	−0.228	0.0511	94.1	−4.45	<0.001	[−0.328, −0.127]		
LSNS-6	Intercept	2.310	0.1206	78.3	19.15	<0.001	[2.074, 2.547]	0.010	0.668
	Time	0.116	0.0574	93.4	2.02	0.047	[0.00321, 0.228]		

The estimates, standard errors, degrees of freedom, t-values, *p*-values, and confidence intervals are presented for each fixed effect (intercept and time) across three different models.

## 4 Discussion

The primary objectives of this research were to document and describe the implementation of the OD-approach by the means of patients’ and their social network members’ rating of OD-network meetings and to assess the clinical outcomes for patients and families receiving treatment based on the OD approach in Italian MHDs over a span of 12 months.

517 OD network meetings involving 58 patients and their social network took place across a span of 12 months. Within these 12 months patients attended an average of eight OD network meetings, and each session saw participation from an average of one social network member, where male patients had a 17% higher number of social network participation in comparison to female patients.

The evaluation of OD-Network meetings using the Session Rating Scale (SRS) and Outcome Rating Scale (ORS) provided evidence of the positive reception (SRS) and perceived effectiveness (ORS) of the Open Dialogue approach. The SRS results indicated that both patients and their social network members consistently rated the sessions highly, with scores significantly above the cross-cultural mean documented cross-cultural examination of the scale by Hafkenscheid et al. (2010). This suggests a high level of satisfaction with the network meetings, reflecting strong therapeutic alliances and effective engagement of participants. Moreover, the linear mixed model analysis of SRS scores revealed that later sessions were rated more positively, indicating a growing appreciation for the meetings as therapy progressed. In contrast, the ORS assessments highlighted a divergence in perceptions of outcomes between patients and their social network members, with patients generally rating the outcomes lower than their social network members. However, there was a positive trend in ORS scores over time, suggesting that both patients and social network members perceived improvements as the therapy continued. One possible hypothesis for the initially higher scores given by family members is that network meetings provide immediate relief by offering support and a sense of being heard, which alleviates their sense of isolation. In contrast, the impact on the well-being of the patient in crisis may take longer to manifest, as the therapeutic process needs time to unfold and address deeper issues. Overall, these findings underscore the value of using both scales to capture different dimensions of the therapeutic experience.

With regard to the effectiveness of the Open Dialogue (OD) approach in enhancing mental health outcomes within the Italian context, this study documents clear clinical improvements across several key indicators. Over a 12-month period, the application of OD principles in network meetings correlated with significant positive changes in the Global Assessment of Functioning (GAF), Clinical Outcomes in Routine Evaluation (CORE-OM), and the Lubben Social Network Scale (LSNS). These findings are particularly noteworthy given the diverse and comprehensive measures employed to assess therapeutic progress.

The use of a linear mixed-effects model provided robust insights into the longitudinal data, revealing a substantial effect of time on all assessed outcomes. Notably, GAF scores showed a significant increase, suggesting improved psychological, social, and occupational functioning among participants. Similarly, CORE-OM scores indicated a decrease in psychological distress and an enhancement in well-being, which aligns with the core objectives of OD in promoting recovery through dialogue and network involvement. Additionally, LSNS scores demonstrated an increase, reflecting strengthened social networks and support systems, which are vital for sustainable mental health recovery.

These findings underscore the potential of the Open Dialogue approach to not only facilitate immediate improvements in mental health conditions but also to contribute to long-term wellness and social integration. The positive trajectory of these clinical outcomes over the study period highlights the value of incorporating network-based, dialogic practices in mental health services, particularly within systems like Italy’s National Health Service that emphasize community-based care.

### 4.1 Comparison with previous research on Open Dialogue

Overall, this study confirms the feasibility of integrating Open Dialogue into the mental health services of Italy, showcasing its adaptability beyond its original implementation in Lapland as evidenced by analogous research conducted in diverse settings (Kłapciński and Rymaszewska, 2015; Gordon et al., 2016; Kinane et al., 2022). Contrary to other healthcare systems where fragmentation (Heumann et al., 2023), diagnosis-specific services (Kinane et al., 2022), and limitation in the costs covered by insurance (Gordon et al., 2016) have been identified as significant impediments, the Italian model distinctly facilitates this approach. In fact, the Italian mental health system, characterized by community-based services that deliver continuous therapeutic support, employs a trans-diagnostic approach within a universally accessible public framework devoid of insurance-based constraints.

It is crucial to highlight that in this study, both outcome and process data collection commenced only after the participating teams had undergone a year of foundational training with expert Finnish trainers and had demonstrated satisfactory fidelity to the organizational and dialogical principles (Pocobello and el Sehity, 2017; Ciliberto et al., 2017; Pocobello, 2021). Fidelity and adherence assessments during network meeting analyses were conducted using unpublished scales that are based on the principles outlined by Olson et al. (2014). These scales, as reported also by Kinane et al. (2022), not only facilitated the evaluation of adherence and fidelity but also significantly aided in the reflection and improvement processes within the teams. Such evaluations are not merely beneficial—they are essential, as both a literature review (Freeman et al., 2019) and an international survey (Pocobello et al., 2023) have underscored the profound challenges of adopting Open Dialogue with full fidelity to its foundational principles across diverse services.

The findings reported in this article suggest that Open Dialogue network meetings are associated with positive clinical outcomes, including reductions in psychological distress, improved overall functioning, and enhanced social networks. These outcomes align with those reported by Seikkula et al. (2011) in Lapland, though there are notable differences in the study populations and methodologies. Unlike Seikkula et al., who focused on patients experiencing initial psychotic episodes, our study included a more diverse sample across a shorter timeframe of one year. Similar improvements have also been reported in pilot studies in the US (Gordon et al., 2016) and the UK (Kinane et al., 2022), where significant enhancements in well-being and functioning were observed.

Patients and their families consistently reported high levels of satisfaction with both the individual therapy network meetings and the overall treatment outcomes, mirroring findings from earlier research in Lapland, which correlated positive clinical outcomes with high satisfaction rates when engaging the entire social network in treatment (Seikkula et al., 2001). Similar positive outcomes in patient and social network satisfaction have been observed in studies outside of Lapland. For example, a study by Gidugu et al. (2021) in the United States also reported high appreciation levels from both patients and families, highlighting the distinctive benefits of Open Dialogue, particularly its emphasis on social network involvement, transparency, respectfulness, and collaborative nature. Additionally, in their study in the UK, Kinane et al. (2022) reported that Peer Supported Open Dialogue received a notably high score of 9.19, which is significantly higher than the score of the same Trust (6.51) and the national average (7.03). These findings collectively suggest that OD effectively meets the expectations and needs of patients and their families within the mental health care context.

In our study, the annual frequency of network meetings was notably lower, with 517 meetings recorded, compared to the 467 meetings reported by Kinane et al. (2022) over a six-month period. This variation may be attributed to a lower threshold for service access in the Italian context, potentially indicating that some patients presented with less severe clinical conditions than those observed in the British study. Concerning social network participation, our findings showed greater involvement in Italy than in the UK, with social network participation accounting for 69.4% of the meetings, compared to 52.5% in the UK. These differences in social network participation could be influenced by several factors, including the prominent role of families in Italian culture and a well-established systemic tradition in mental health care.

### 4.2 Implications for the implementation of Open Dialogue

This study confirms the feasibility of integrating the Open Dialogue (OD) approach within Italian mental health departments. It demonstrates that professionals can be effectively trained and equipped to adopt this innovative model in a community mental health system ideally suited for OD. Notably, department directors interviewed before the implementation recognized OD’s compatibility with the Basaglia Reform, viewing it as a means to “relaunch” it (Pocobello, 2021). In particular, services such as those in Trieste had already aligned with the first five organizational principles of OD prior to its introduction. Therefore, the training focused primarily on the dialogic principles of dialogism and tolerance of uncertainty—relatively novel concepts across these services, which became the central themes of the training and supervision sessions (Pocobello and el Sehity, 2017).

Furthermore, the positive outcomes observed suggest that OD offers tangible benefits to patients within the Italian mental health system and is highly valued by both patients and their families. Its successful implementation in diverse urban and rural contexts also underscores the potential for scaling the OD approach across the country.

However, the long-term effectiveness of OD depends crucially on sustained monitoring and supervision. Although the project demonstrated effective management of fidelity and adherence, the end of the project introduces a risk of standards slipping without continuous oversight. This underscores the urgency of establishing permanent mechanisms to ensure that high standards of OD practice are maintained over the long term.

Overall, this study makes a compelling case for considering OD as a valuable addition to existing mental health practices in Italian healthcare settings, encouraging further exploration and integration of this model into routine care protocols.

Among the lessons learned from the Italian OD program, extensive training in OD with expert trainers seemed crucial for successful implementation. The selection process, where departments chose candidates based on voluntary participation and intrinsic motivation, appeared effective in ensuring that those trained were genuinely committed to the OD approach. This commitment seems essential for the sustainability and fidelity of OD practices. Future implementations might benefit from continuing to prioritize voluntary and motivated participation in training programs.

The project also highlighted several systemic challenges, including the need for consistent funding, administrative support, and alignment with national health policies. Addressing these challenges could be crucial for the broader implementation of OD. Policymakers and health administrators might need to recognize the value of OD and allocate resources to support its integration into mental health services.

Furthermore, research has appeared fundamental in addressing the challenges encountered during implementation. It has played a key role in promoting the quality of the intervention and fostering a reflective attitude in both clinical practice and implementation processes. Research has also been important for maintaining the network of services and creating a professional network that has extended well beyond the initial project timeframe.

### 4.3 Limitations and future directions

This study has several notable limitations. First, the relatively small sample size of 58 participants may limit the generalizability of the findings. Additionally, the sample size shrunk over time due to attrition, which could introduce bias and affect the robustness of the results.

The absence of a control group makes it difficult to definitively attribute the observed changes at the three time points to the Open Dialogue approach rather than to natural progression over time. Furthermore, the 12-month follow-up period may be too brief to fully capture the long-term effects and sustainability of the improvements. This underscores the need for extended monitoring to more accurately assess the durability of the outcomes.

Another limitation is the use of the Session Rating Scale (SRS) and Outcomes Rating Scale (ORS). Although these scales are widely used in clinical settings, their application in research may be considered a limitation due to potential biases and the subjective nature of self-reported data. However, these scales also offer a significant strength to the study. They effectively capture the experiences of end users, providing valuable insights into client satisfaction and the therapeutic relationship.

Looking ahead, future research should focus on long-term, large-scale longitudinal studies to better understand the sustained impacts of OD. Moreover, there is a significant gap in cost-effectiveness analyses, which are essential for evaluating the economic viability and potential for broader application of OD.

## 5 Conclusion

The findings of this study reflect a significant affirmation of the Open Dialogue (OD) approach within the Italian mental health service context, underscoring its potential as a transformative model for mental health care. The consistent improvements in Global Assessment of Functioning (GAF), Clinical Outcomes in Routine Evaluation (CORE-OM), and the Lubben Social Network Scale (LSNS) over the 12-month period demonstrate the effectiveness of OD in enhancing psychological well-being, social functioning, and network support among participants.

The study highlighted the value of the OD approach in fostering substantial client and family engagement, which is crucial in mental health recovery. The Session Rating Scale (SRS) and Outcome Rating Scale (ORS) evaluations illustrated high satisfaction levels and perceived positive outcomes, reinforcing the relational and collaborative foundation of OD. These positive evaluations from clients and their networks not only validate the approach but also illustrate its capacity to create a supportive and effective therapeutic environment.

Looking to the future, these results suggest that integrating OD principles into broader mental health services could substantially improve care outcomes. The emphasis on immediate, flexible, and continuous care, in alignment with individual needs and involving a support network, aligns well with current shifts toward more personalized and patient-centered care models in mental health services globally.

Moreover, the successful implementation of OD in Italian MHDs, which shares characteristics with Finland’s public and community-based healthcare system, suggests that this approach can be adapted to diverse health systems with varying resources and cultural contexts. This adaptability is crucial for the expansion of OD and highlights its potential for adoption in other regions seeking innovative and effective mental health solutions, particularly in systems that prioritize public health and community engagement.

In conclusion, the integration of OD into Italian MHDs not only enhances clinical outcomes but also embodies a shift toward more humane, responsive, and effective mental health care. By continuing to foster research, training, and implementation of OD, there is potential for a significant paradigm shift in how mental health care is delivered worldwide. This could lead to systems that not only manage symptoms but also empower individuals and their communities, contributing to a more holistic approach to mental health and well-being.

## Data Availability

The raw data supporting the conclusions of this article will be made available by the authors, without undue reservation.

## Footnotes

^1^ https://www.hopendialogue.net/
